# Serum Sp17 Autoantibody Serves as a Potential Specific Biomarker in Patients with SAPHO Syndrome

**DOI:** 10.1007/s10875-020-00937-w

**Published:** 2021-01-03

**Authors:** Hongqin You, Guanglei Dang, Bichao Lu, Siya Zhang, Chen Li, Lun Wang, Yu Hu, Hui Chen, Jianmin Zhang, Wei He

**Affiliations:** 1 Department of Immunology, Key Laboratory of T Cell Mediated Cancer Immunotherapy, Institute of Basic Medical Sciences, Chinese Academy of Medical Sciences and School of Basic Medicine, State Key Laboratory of Medical Molecular Biology, Peking Union Medical College, Beijing, China; 2 Department of Immunotherapy, Affiliated Cancer Hospital of Zhengzhou University and Henan Cancer Hospital, Zhengzhou, Henan China; 3 Department of Traditional Chinese Medicine, Peking Union Medical College Hospital, Peking Union Medical College and Chinese Academy of Medical Sciences, Beijing, China; 4 Institute of Clinical Medicine, Peking Union Medical College Hospital, Chinese Academy of Medical Sciences & Peking Union Medical College, Beijing, China

**Keywords:** SAPHO syndrome, serum autoantibody, Sp17, biomarker

## Abstract

SAPHO (synovitis, acne, pustulosis, hyperostosis, and osteitis) syndrome shows a wide variability in musculoskeletal and cutaneous manifestations, and it is therefore underrecognized and misdiagnosed in the clinic due to a lack of specific markers. In this study, we aimed to identify specific biomarkers by screening serum autoantibodies in SAPHO patients with a 17K human whole-proteome microarray. The serum anti-Sp17 autoantibody was identified and verified to be a specific biomarker in patients with SAPHO syndrome. Indeed, the level of the anti-Sp17 autoantibody was significantly increased in patients with active SAPHO compared to patients with an inactive disease and healthy controls (*P* < 0.05). Additionally, serum anti-Sp17 autoantibody levels correlated with those of serum hypersensitive C-reactive protein (hsCRP), the erythrocyte sedimentation rate (ESR), and β-crosslaps (β-CTx) in patients with active SAPHO disease. Moreover, anti-Sp17 autoantibody levels were markedly decreased after anti-inflammatory treatment with pamidronate disodium, which downregulated levels of hsCRP and ESR in patients with active SAPHO. Thus, serum levels of the anti-Sp17 autoantibody might serve as a specific biomarker for the diagnosis of SAPHO syndrome or for monitoring the disease status.

**Supplementary Information:**

The online version contains supplementary material available at 10.1007/s10875-020-00937-w.

## Introduction

Synovitis, acne, pustulosis, hyperostosis, and osteitis (SAPHO) syndrome was first proposed by Chamot et al. [1] in 1987 as a rare disease that occurs in 30- to 50-year-old individuals. The predominance of individual clinical manifestations varies among patients with SAPHO syndrome [2]. In 1988, 4 diagnostic criteria for SAPHO syndrome were first proposed by Benhamou [3, 4], including (1) osteoarticular manifestations with polymeric acne and fulminant acne or septic hidradenitis, (2) osteoarticular manifestations with palmar pustulosis, (3) hyperosteogeny (upper chest wall, extremities, or spine) with or without skin lesions, and (4) chronic multifocal recurrent osteomyelitis (CMRO) involving the axial or peripheral bones with or without skin lesions. Patients with one of the 4 conditions listed above can be diagnosed with SAPHO syndrome. SAPHO syndrome is often unrecognized or misdiagnosed due to its challenging diagnosis caused by the wide variability in musculoskeletal and cutaneous manifestations [5, 6].

Bone pain caused by severe osteoarticular lesions is one of the most common symptoms prompting SAPHO patients to visit hospitals. Therefore, in some clinical studies, the visual analogue scale (VAS) score, which evaluated the degree of pain, was used to measure the disease activity of SAPHO [7]. However, pain, especially chronic pain, is influenced by and interacts with physical, psychological, social, and contextual factors. The VAS score does not accurately represent the disease severity. Moreover, the levels of C-reactive protein (CRP) and the erythrocyte sedimentation rate (ESR) are also elevated in most patients, but not in complete accordance with the activity of SAPHO syndrome [8]. Thus, no specific markers have been identified for the diagnosis or monitoring of the disease status of patients with SAPHO syndrome.

Currently, standardized treatment protocols are not available for patients with SAPHO syndrome. Most treatments are empirical and mainly attenuate the pain associated with SAPHO syndrome. Nonsteroidal anti-inflammatory drugs and analgesics are applied as first-line agents. Anti-rheumatic drugs have also exerted beneficial effects on some patients [3, 9]. Because of the inhibition of bone resorption and the anti-inflammatory effect, bisphosphonates are clinically used for palliative treatment of patients with SAPHO syndrome. Osteocalcin and β-crosslaps (β-CTx), which can reflect the bone metabolism, have been suggested as an ideal prognostic marker for bisphosphonates treatments. However, they could not reflect the therapeutic effect of the treatment methods other than bisphosphonates. More predictors of the efficacy of treatment with antibiotics, bisphosphonates, or immunosuppressive drugs are available.

The pathogenesis and etiology of SAPHO syndrome are not yet clear; they may be related to heredity, infection, and immunity [10]. Previous studies have reported multiple dysfunctions of the immune system in SAPHO. For example, according to Nedelec et al. [11], an infectious state contributing to strong humoral and cellular proinflammatory responses may trigger SAPHO syndrome, and the cellular immune response may also be abnormal. Activation of the Th17 axis, but not the Th1 or Th2 axis, has been observed [12]. From the perspective of autoimmunity, several previous studies have assessed anti-thyroid peroxidase (TPO), anti-thyroglobulin (Tg), and anti-nuclear antibodies in patients with SAPHO syndrome [13]. Regardless, these autoantibodies are not specific to SAPHO syndrome and have little significance. Thus, the identification of target autoantigens is important for understanding the etiology of SAPHO syndrome and for the diagnosis and/or monitoring of the disease status.

Multiplex assays have emerged for autoantibody high-throughput screening, enabling the rapid identification of subsets of patients to facilitate diagnostic and predictive medicine [14]. In this study, a 17K whole-genomic protein microarray was applied to screen the profile of serum autoantibodies in patients with SAPHO syndrome to identify specific biomarkers for the diagnosis or disease status monitoring.

## Materials and Methods

### Patients and Healthy Controls

Healthy controls (HC), patients with SAPHO syndrome, patients with systemic lupus erythematosus (SLE), and patients with rheumatoid arthritis (RA) were recruited from the Peking Union Medical College Hospital (PUMCH). The committees of both the PUMCH and Chinese Academy of Basic Medical Science approved the use of clinical samples for this project (identifier: ZS-944). The subjects meeting the diagnostic criteria proposed by Benhamou [4] for SAPHO syndrome were included in this study. The exclusion criteria are as follows: (1) women in pregnancy or lactation, (2) septic osteomyelitis, (3) infectious chest wall arthritis, (4) infections PPP, (5) palmoplantar keratodermia, (6) DISH except for fortuitous association, (7) osteoarticular manifestations of retinoid therapy. The patients with SLE and RA fulfilled the American College of Rheumatology (ACR) criteria for SLE [15] or RA [16], respectively, but did not meet the criteria for SAPHO syndrome. The demographic characteristics, osteoarticular symptoms, skin manifestations, and lesion sites of patients with SAPHO syndrome on bone scintigraphy were recorded. Laboratory evaluation included erythrocyte sedimentation rate (ESR, 0–15 mm/h for male and 0–20 mm/h for female), hypersensitive C-reaction protein (hsCRP, 0–3 mg/L), β-CTx (0.21–0.44 ng/mL), and osteocalcin (1.8–8.4 ng/mL) that were also collected. Serum samples were collected, centrifuged at 1000×*g* for 10 min, aliquoted, and stored at − 80 °C until use.

### Protein Microarray Profiling

17K HuProt™ human whole-proteome microarray slides (CDI, USA) were initially blocked with 3% BSA-TBST buffer at room temperature (RT) for 1 h. The sera from every 5 patients or HCs were mixed as the primary antibody and incubated with the arrays for 1 h at RT. TBST buffer was used to wash the arrays five times. The secondary antibody, a Cy3-conjugated goat anti-human IgG antibody (Biolegend, USA), was diluted and incubated with the arrays for 1 h at RT. The arrays were washed as described above. The primary antibody against the positive control, i.e., a rabbit anti-GST monoclonal antibody (CST, USA), was diluted and incubated with the same microarray slides at RT for 1 h. The slides were washed as described above. The secondary antibody for the positive control, Cy5-labeled goat anti-rabbit IgG (Biolegend, USA), was diluted 1:500 and incubated with the array for 1 h at RT, followed by washing as described above. Three independent protein chip tests were performed, and the chips were scanned using a GenePix 4000B fluorescence microarray scanner (CapitalBio, China). The protein chip data were processed by GenePixPro 5.1 software. The mean value of duplicates was used for data analysis. The signal-to-noise ratio at a wavelength of 532 nm (SNR532) and the ratio of 532 nm to 635 nm (see below) were used for the quantitative analysis of protein spots. The specific equations used for the calculations are as follows: SNR532 = (mean foreground at 532 nm − mean background at 532 nm)/(standard deviation of the background at 532 nm); ratio = (mean foreground at 532 nm − mean background at 532 nm)/(mean foreground at 635 nm − mean background at 635 nm).

The criteria for choosing candidates were as follows: points with SNR532 values over 2, which were found in SAPHO patients but not in HCs, were considered positive points.

### Plasmids, Recombinant Proteins, and Gene Cloning

The plasmid used for *Sp17* overexpression in eukaryotic cells was constructed with the Gateway Cloning System (Invitrogen, USA) according to the manufacturer’s instructions. An alternate Sp17 plasmid was constructed for *Escherichia coli* expression by cloning the full-length sequence of the *Sp17* gene into the PET-30a easy vector and transforming it into *E. coli* BL-21(DE3) (TransGen, China). Correct construction of the plasmid was confirmed by DNA sequencing. BL-21 cells were induced with isopropyl β-D-1-thiogalactopyranoside (IPTG; 1 mmol/L; Sigma) at 37 °C for 6 h, and the recombinant Sp17 protein was purified using prepacked HisTrap high-performance columns (GE Health, USA).

To construct a *UACA* overexpression plasmid, the full-length gene sequence was purchased (Sino Biological, China) and cloned into the cFUGW vector using Phusion DNA polymerase (Biolabs, USA). The experimental procedures were performed according to the manufacturer’s instructions. The correct plasmid was transfected into 293 T cells using Lipofectamine 2000 (Invitrogen, USA).

### Western Blotting Analysis

In western blotting studies, 20 μg per lane whole-cell lysate was separated by SDS-PAGE, and the proteins were transferred to NC membranes. The NC membranes were cut into different strips for incubation with sera from different HCs or SAPHO syndrome patients. The primary antibody, sera from HCs or SAPHO syndrome patients, was diluted at 1:100 and incubated at 4 °C overnight. The secondary antibody, goat anti-human IgG (Thermo Fisher, USA), was diluted 1:5000 and incubated with the membrane for 2 h at RT. The NC membranes were combined in imaging steps. Chemiluminescent horseradish peroxidase (HRP) substrate (Pierce, USA) was added to the spliced NC membrane, followed by detection using a chemiluminescence imaging analysis instrument (Clinx, China).

### Enzyme-Linked Immunosorbent Assay

Serum total IgG (Abcam, USA) and UACA autoantibody levels (CUSABIO, China) were assessed according to the manufacturer’s instructions.

For the Sp17 autoantibody test, His-tagged recombinant Sp17 (0.2 μg/mL) was coated onto 96-well test plates. Wells without antigen coating were used as blank controls. The plates were washed and blocked with 5% BSA-PBST. Serum samples, as the first antibody, were diluted at 1:300. Wells with anti-His antibody were used as a positive control. Blank wells did not include any reagents, and negative control wells contained phosphate-buffered saline (PBS). The plates were incubated at 37 °C for 1 h and then washed 5 times. HRP-labeled anti-human IgG (Thermo Fisher, USA) was diluted and incubated at 37 °C for 1 h. TMB was added after 5 washes, and the reaction was terminated by adding H_2_SO_4_ (0.2 mol/L). Absorbance at OD450 was measured using a microplate reader (Thermo Fisher, USA).

### Statistical Analysis

We performed statistical analysis using SPSS 19.0 software (IBM Corp., USA). Independent samples *t* tests were employed to compare means between the two groups. Analysis of variance (ANOVA) was used for three or more sets of data. Spearman correlation was applied to analyze correlations. The chi-squared test (or Fisher’s exact test if required) was used for categorical variables. Means between different treatment cycles were compared with paired *t* tests.

## Results

### Clinical Characteristics

The demographic characteristics of patients with SAPHO syndrome, patients with SLE, and patients with RA and HCs were recorded (Table 1). No significant differences in age (*P* = 0.228) and sex (*P* = 0.055) were observed among the four groups. Pain in the anterior chest wall, the most characteristic osteoarticular symptom of SAPHO syndrome, occurred in all 73 patients with SAPHO syndrome. The second most frequently reported symptomatic site was axial bone and joint pain (47.9%), and symptoms related to peripheral bone and joint pain were reported by 26.0% of the patients. Regarding skin manifestations, most patients (84.9%) had palmoplantar pustulosis (PPP). Severe acne (SA) and psoriasis vulgaris (PV) were present in 13.7% and 19.2% of the patients, respectively. On whole body bone scintigraphy, 91.8% of the patients exhibited increased tracer uptake in the sternocostoclavicular region, consistent with the symptoms. The second most frequently affected site in the axial skeleton was the vertebrae (49.3%), followed by the sacroiliac joints (27.4%). Only 15.1% patients presented lesions in peripheral bones and joints. Involvement of the craniofacial bone and joints was observed in 4.1% of patients.

**Table 1:** Clinical characteristics of patients and HCs

Demographic characteristics				
**SAPHO**		**SLE**	**RA**	**HC**
Sex, female/age (years)	43 (47.9 ± 9.9)	9 (35 ± 10.6)	13 (49.8 ± 11.2)	17 (46.0 ± 12.8)
Sex, male/age (years)	30 (39.9 ± 7.7)	3 (44 ± 22.6)	3 (49.7 ± 10.6)	16 (44.8 ± 12.3)
**Osteoarticular symptoms**		**General symptoms**	**General symptoms**	
Axial bones and joints pain	35/73 (47.9%)	Cardiovascular system 8/12 (66.7%)	Progression year ≥ 5 4/14 (28.6%)	
Anterior chest wall pain	73/73 (100%)	Central nervous system 4/12 (33.3%)	Tender joints ≥ 10 8/14 (57.1%)	
Peripheral bone and joint pain	19/73 (26.0%)	Musculoskeletal system 6/12 (50%)	VAS ≥ 4 9/14(64.3%)	
**Skin manifestations**		Dermatological 4/12 (33.3%)		
PPP	62/73 (84.9%)	Gastrointestinal system 4/12 (33.3%)		
SA	10/73 (13.7%)	Respiratory system 4/12 (33.3%)		
PV	14/73 (19.2%)	Genito-urinary system 10/12 (83.3%)		
**Lesion sites on bone scintigraphy**		**Serology tests**	**Serology tests**	
Sternocostoclavicular region	67/73 (91.8%)	ESR (36.7 ± 31.4) mm/h	CRP (30.3 ± 18.1) mg/L	
Sacroiliac joint	20/73 (27.4%)	CRP (12.3 ± 24.3) mg/L	Anti-CCCP antibody 14/14 (100%)	
Vertebrae	36/73 (49.3%)	ANA 12/12 (100%)	Anti-RF (IgM) 4/14 (28.6%)	
Peripheral bones and joints	11/73 (15.1%)	Anti-ds DNA 8/12 (66.7%)	Anti-RF (IgG) 12/14 (85.7%)	
Craniofacial bone and joints	3/73 (4.1%)	Anti-SS-A 6/12 (50%)		

Data are presented as means ± SD or numbers of patients with the corresponding characteristics/total number of patients (%) unless otherwise indicated

*PPP* palmoplantar pustulosis, *SA* severe acne, *PV* psoriasis vulgaris

### Anti-Sp17 and Anti-UACA Autoantibodies Were Identified in Sera from Patients with SAPHO Syndrome Using the 17K Human Whole-Proteome Microarray

**Fig. 1:**
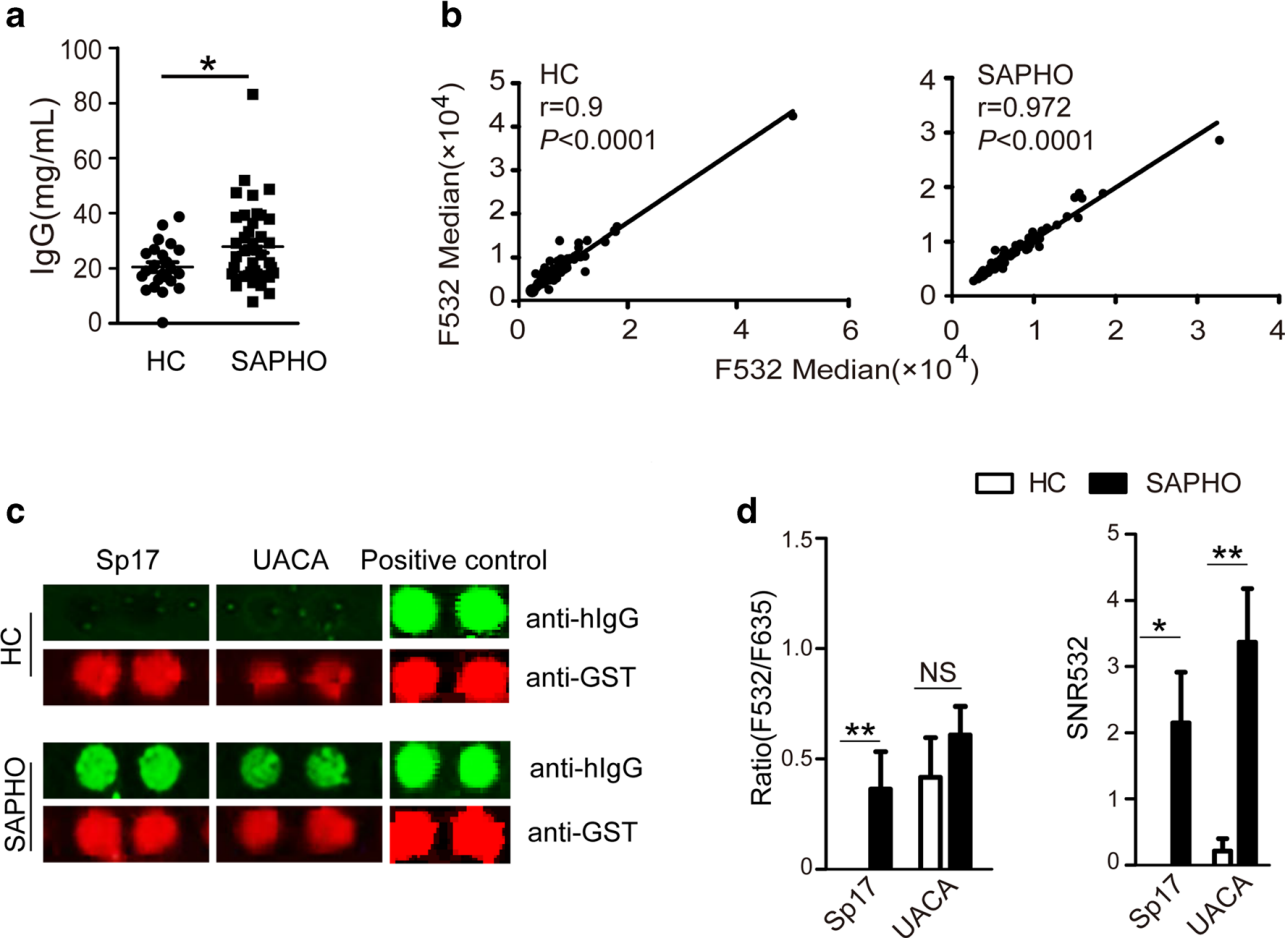
Autoantibodies against Sp17 and UACA were identified by screening the profile of autoantibodies in the sera of SAPHO patients with a 17K human proteome microarray. **a** Total serum IgG levels were increased in patients with SAPHO syndrome (*n* = 43) compared with HCs (*n* = 21). **b** Analysis of the consistency of the fluorescence intensity of duplicate protein chip experiments using serum samples from HCs and patients with SAPHO syndrome. F532 median: the median fluorescence intensity at 532 nm. **c** Cropped images of autoantibodies against Sp17 and UACA on protein chips. Green fluorescence represents the results of serum autoantibody signals; red fluorescence produced by the anti-GST antibody represents protein loading. **d** Quantitative analysis of the fluorescence intensities of anti-Sp17 and anti-UACA autoantibodies in HCs and SAPHO patients. F532: the signal intensity detected from serum autoantibodies. F635: the signal intensity detected from anti-GST antibodies. SNR signal-to-noise ratio. *P < 0.05; **P < 0.01; NS no significance

We first assessed total IgG levels in the sera from 43 patients with SAPHO syndrome and 21 HCs. The results showed significantly higher levels of total IgG in the sera of SAPHO syndrome patients than in HCs (Fig. 1a). Next, we screened the profile of autoantibodies in SAPHO patient sera by using 17K human whole-proteome microarrays (containing 17,000 human proteins) [17]. Correlations of median fluorescence intensity at 532 nm between duplicate spots were plotted. The screening results showed excellent reproducibility (Fig. 1b). Through differential analysis of the protein chip, two autoantibodies, against Sp17 and UACA, were identified only in the sera from patients with SAPHO syndrome and not in sera from HCs (Fig. 1c). Further quantitative analysis of SNR532 values and ratios at 532 nm confirmed the above observation (Fig. 1d). These findings warrant further investigation in a larger sample set of individuals.

### Anti-Sp17 Autoantibodies Were Detected in Sera from Patients with SAPHO Syndrome

**Fig. 2:**
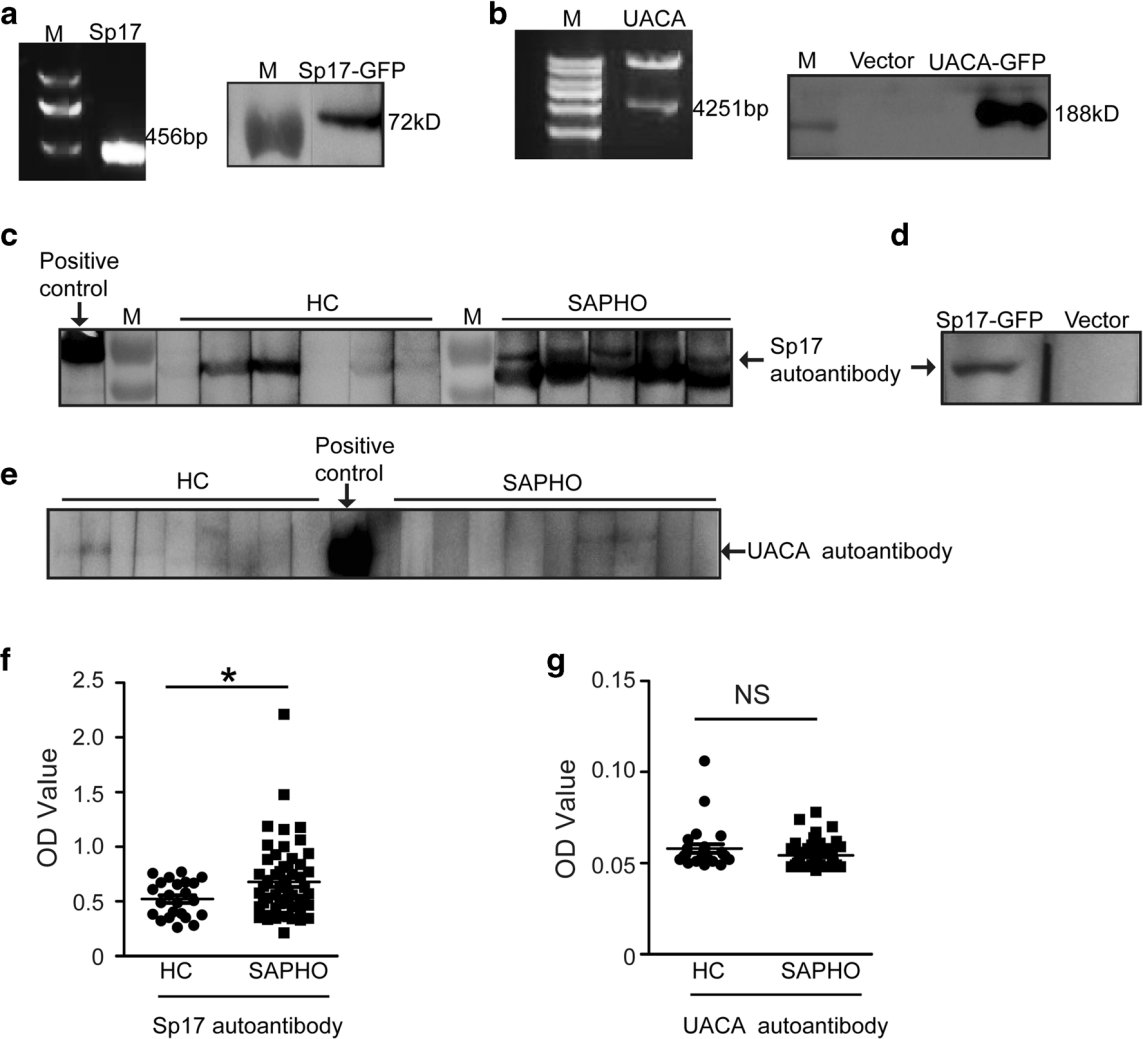
Anti-Sp17 autoantibodies were significantly elevated in the sera of patients with SAPHO syndrome. **a** Recombinant Sp17-GFP fusion proteins were overexpressed in 293 T cells. The left panel represents the expected 456-bp DNA fragment after restriction enzyme digestion of *Sp17* overexpression plasmids. The right panel represents immunoblot analysis for the identification of Sp17-GFP fusion proteins by using anti-GFP mAb. **b** Recombinant UACA-GFP fusion proteins were overexpressed in 293 T cells. The full-length *UACA* gene was detected at 4251 bp. UACA-GFP fusion proteins at 188 kDa were verified using anti-UACA antibodies. M represents the molecular weight marker. **c** Representative immunoblot showing the serum levels of Sp17 autoantibodies from 6 of the 22 HCs and 5 of the 30 patients with SAPHO syndrome. Positive control: anti-GFP antibody. The NC membrane was cut into strips for incubation with sera from different HCs or SAPHO syndrome patients. **d** Western blot for verifying the binding of the serum Sp17 autoantibody with the transfected Sp17 protein. **e** Representative immunoblot showing the serum levels of UACA autoantibodies in 9 of the 28 HCs and 10 of the 30 patients with SAPHO syndrome. Positive control: anti-GFP antibody. **f** Indirect ELISA analysis of serum Sp17 autoantibodies in HCs (*n* = 33) and patients with SAPHO syndrome (*n* = 40). **g** ELISA analysis of serum UACA autoantibody levels of HCs (*n* = 33) and patients with SAPHO syndrome (*n* = 40). Independent samples *t* tests were used to compare means between healthy controls and patients. *P < 0.05; **P < 0.01; NS no significance

To further verify whether Sp17 and UACA autoantibodies are present in a larger sample of SAPHO sera, Sp17 and UACA were overexpressed in 293 T cells (Fig. 2a and b), and western blot analysis with SAPHO patient sera confirmed the presence of anti-Sp17 autoantibodies (Fig. 2c). The positivity rate of serum Sp17 autoantibodies in patients with SAPHO syndrome was significantly higher than in HCs as shown in Table 2. In addition, the specific interaction between the anti-Sp17 autoantibody and overexpressed Sp17 was confirmed by western blotting using cell lysates from empty vector- or Sp17-transfected cells (Fig. 2d). Unfortunately, no autoantibodies for UACA were detected in the sera from SAPHO patients by western blot analysis (Fig. 2e).

**Table 2:** Proportions of autoantibody-positive samples

		Total samples (*N*)	Positive samples (*N*)	Proportion	Chi-squared value	*P* value
Anti-Sp17	HC	22	6	27%	14.174	< 0.001
	SAPHO	30	25	83%		
Anti-UACA	HC	28	0	0		
	SAPHO	30	0	0		

A *P* value < 0.05 is considered statistically significant

Next, we applied indirect ELISA to verify the presence of autoantibodies as well as to measure the levels of serum autoantibodies from SAPHO patients and HCs reacting with Sp17 and UACA. The ELISA results exhibited substantial consistency with the western blot analysis. Only anti-Sp17 autoantibodies were detectable in the sera from patients with SAPHO syndrome, and levels higher than those from HCs (Fig. 2f). However, the levels of UACA autoantibodies in the sera of both SAPHO patients and HCs were lower than the detection limit of the ELISA kit (Fig. 2g). Taken together, these results confirm that anti-Sp17 autoantibodies are elevated in the sera of patients with SAPHO syndrome.

### Serum Levels of Sp17 Autoantibodies Were Only Elevated in Patients with Active SAPHO Syndrome

**Fig. 3:**
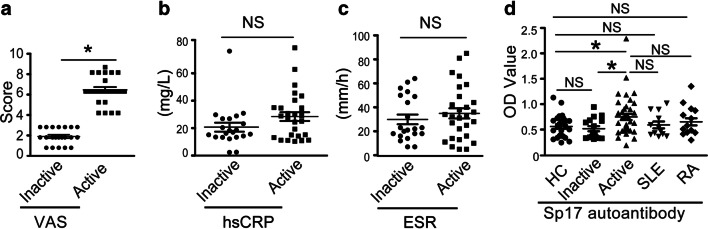
Levels of serum Sp17 autoantibodies were significantly elevated in SAPHO patients with active disease. **a** Comparison of VAS scores in active and inactive SAPHO groups. VAS scores were assessed at a PUMCH visit. **b** Serum hsCRP levels of SAPHO patients with inactive and active disease. **c** ESR of SAPHO patients with inactive and active disease. hsCRP level or ESR was tested in all patients when they visited the PUMCH. **d** Comparison of serum Sp17 autoantibody levels in HCs (*n* = 30), patients with inactive (*n* = 24) and active (*n* = 26) SAPHO disease, patients with SLE (*n* = 13), and patients with RA (*n* = 9). *P < 0.05. NS no significance

To further explore the clinical significance of serum Sp17 autoantibodies in SAPHO syndrome, we collected the clinical data of SAPHO patients and HCs. First, VAS scores were selected as the primary measure of disease activity, with hsCRP and ESR as secondary supplemental indicators. Based on the similarity in symptoms with ankylosing spondylitis, SAPHO patients with a score for spinal pain of 4 cm or more on a 10-cm visualanalogue scale (with higher numbers indicating greater disease activity) were active, others were considered to be inactive groups according to previous study [18] (Fig. 3a). Levels of hsCRP and ESR were also slightly higher in patients with active SAPHO than in patients with inactive disease, but no significant difference was noted (Fig. 3b and c). Notably, serum levels of Sp17 autoantibodies were only increased in patients with active SAPHO disease. Furthermore, compared with patients with SLE or RA, serum levels of Sp17 autoantibodies in patients with active SAPHO syndrome were also slightly higher, indicating that elevated serum Sp17 autoantibody levels occurred only in patients with active SAPHO syndrome (Fig. 3d). In summary, elevation of the levels of serum Sp17 autoantibodies in patients with active SAPHO indicates that it may be associated with disease status.

### Serum Levels of Sp17 Autoantibodies in Patients with Active SAPHO Syndrome Exhibited Good Consistency with Systemic Inflammation Indices

**Fig. 4:**
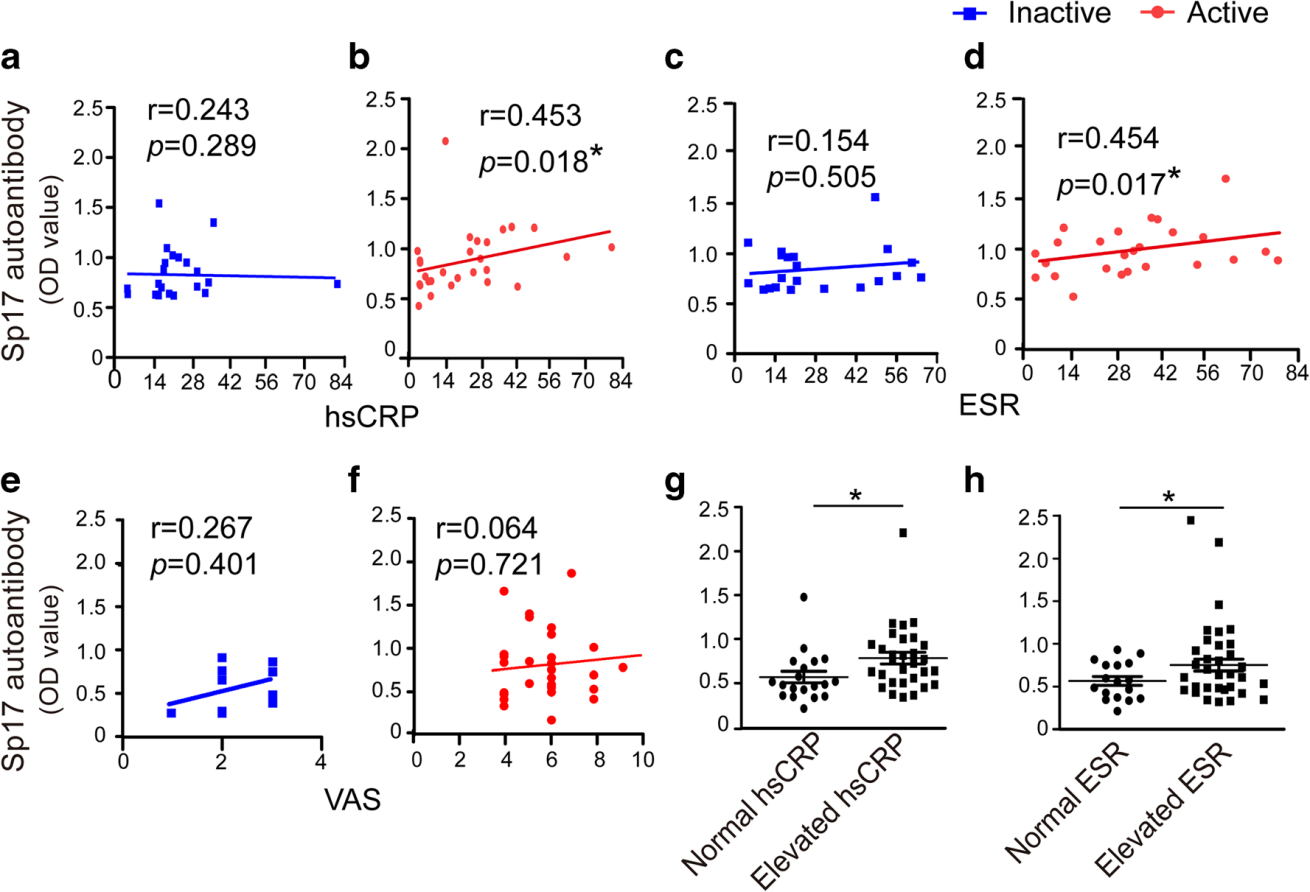
Levels of serum Sp17 autoantibodies in patients with active SAPHO syndrome had good consistency with systemic inflammation indices. Correlations between levels of serum Sp17 autoantibodies and hsCRP concentrations (**a** and **b**), ESRs (**c** and **d**), and VAS (**e** and **f**) were analyzed in SAPHO patients with inactive (*n* = 24) and active (*n* = 26) disease. **g** Comparison of levels of serum Sp17 autoantibodies in normal (*n* = 20) and elevated (*n* = 30) hsCRP groups. **h** Comparison of serum levels of Sp17 autoantibodies in normal (*n* = 17) and elevated (*n* = 33) ESR groups. Independent samples *t* tests were used to compare means between the two groups. The Spearman correlation was used to analyze correlations. *P < 0.05. NS no significance

We next assessed the correlations between levels of serum Sp17 autoantibodies and various indicators of SAPHO syndrome. In patients with inactive SAPHO syndrome, no significant correlations were observed between levels of serum Sp17 autoantibodies and hsCRP (Fig. 4a), ESR (Fig. 4c), and VAS (Fig. 4e). In SAPHO patients with active disease, we noted that the levels of Sp17 autoantibodies were significantly and positively correlated with hsCRP (Fig. 4b) and ESR (Fig. 4d), but no significant correlation with VAS (Fig. 4f) was noted. We also found that the patients with elevated hsCRP (Fig. 4g) or serum ESR (Fig. 4h) had higher levels of serum Sp17 autoantibodies, indicating that levels of serum Sp17 autoantibodies correlated closely with the inflammatory status in SAPHO patients.

### Serum Levels of Sp17 Autoantibodies Were Correlated Closely with the Bone Metabolism Status in Patients with Active SAPHO Syndrome

**Fig. 5:**
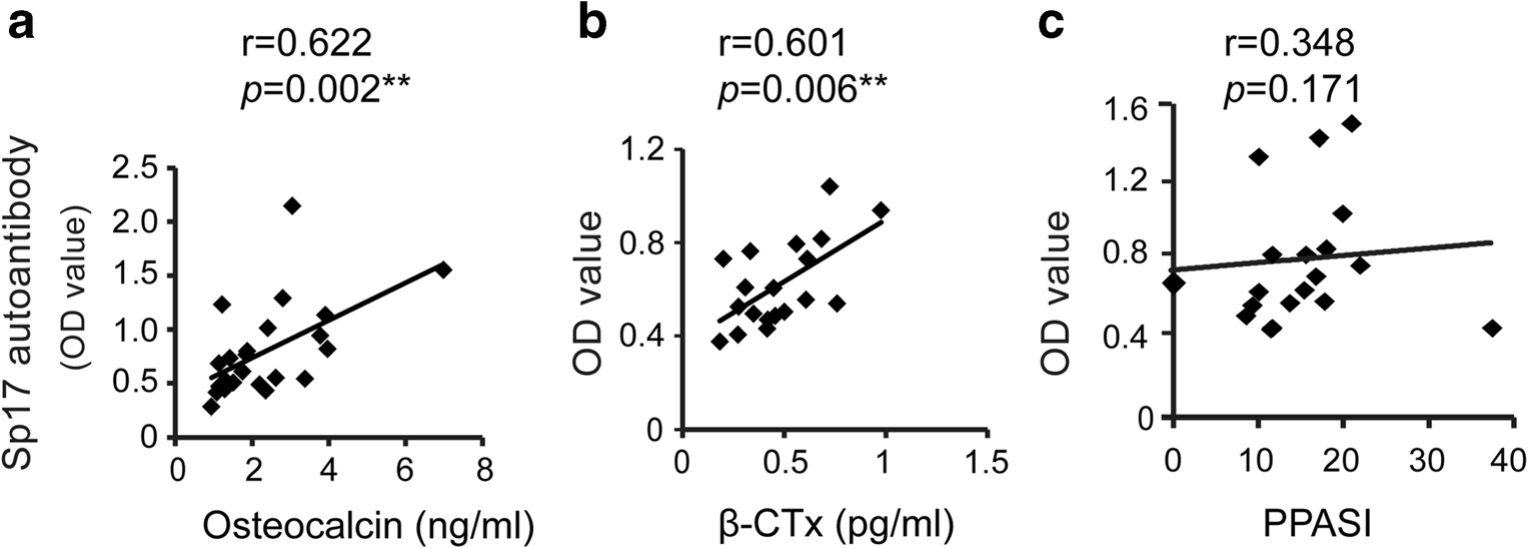
Correlations between levels of serum Sp17 autoantibodies and osteocalcin concentrations (*n* = 23) (**a**), β-CTx concentrations (*n* = 23) (**b**), and PPASI (*n* = 17) (**c**) were analyzed. PPPASI is a comprehensive score calculated by a specific formula according to the severity and area of skin lesions in SAPHO patients and used to assess the severity of PPP. The Spearman correlation was used to analyze correlations. **P < 0.01. NS no significance

We wonder whether there was a correlation between levels of serum Sp17 autoantibodies and specific features of active SAPHO patients. Bone joint pain caused by osteoarthritis and osteolytic leision and palmoplantar pustulosis (PPP) were the most common bone and skin manifestations respectively of SAPHO patients included in this study. Thus, we firstly analyzed the relationships of serum Sp17 autoantibodies levels with serum osteocalcin and β-CTx concentrations. Results showed that the levels of Sp17 autoantibodies were significantly and positively correlated with osteocalcin (Fig. 5a) and β-CTx concentrations (Fig. 5b), but no significant correlation with palmoplantar pustulosis area and severity index (PPPASI) (Fig. 5c) was noted. Above results showed that serum levels of Sp17 autoantibodies correlated closely with the bone metabolism status in SAPHO patients.

### Serum Levels of Sp17 Autoantibodies in Patients with Active SAPHO Syndrome Were Significantly Decreased After Pamidronate Disodium Treatment

**Fig. 6:**
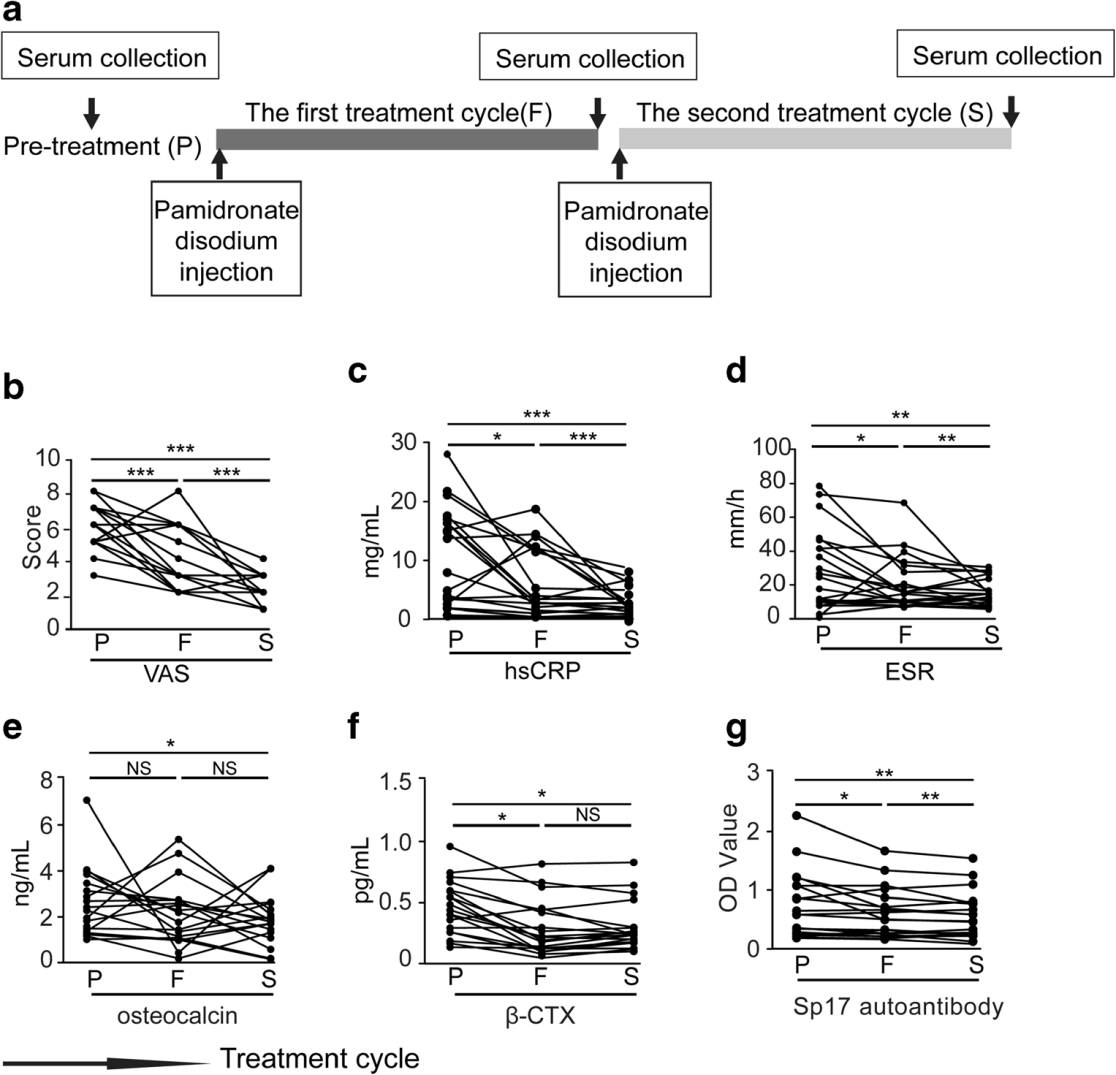
Levels of serum Sp17 autoantibodies in SAPHO patients with active disease were significantly decreased after treatment with pamidronate disodium. **a** Schematic chart of sample collection. The black arrows indicate the time points of serum collection and treatment with pamidronate disodium injection. Patients with active SAPHO syndrome (*n* = 19) were treated with pamidronate disodium for two treatment cycles once every 3 months. Pretreatment blood samples were obtained before the first treatment cycle. Patients were subjected to treatment with pamidronate disodium (1 mg/kg, IV, QD) on the first 3 days of each treatment cycle. After each treatment cycle, blood samples were collected again. **b**–**g** VAS scores and levels of hsCRP, ESR, osteocalcin, β-CTx, and Sp17 autoantibodies at pretreatment (P), after the first treatment cycle (F), and after the second treatment cycle (S). NS not significant. *P < 0.05, **P < 0.01, ***P < 0.001

Finally, we investigated the effect of pamidronate disodium treatment on levels of serum Sp17 autoantibodies in SAPHO patients. Pamidronate disodium, an inhibitor of bone resorption, is used primarily in the management of tumor-induced hypercalcemia and Paget’s disease of the bone. A detailed time chart of the treatment plan is shown in Fig. 6a. VAS scores decreased significantly after each treatment cycle and were less than 4 at the earliest time point after treatment (Fig. 6b), suggesting low disease activity or remission. Additionally, levels of hsCRP and ESR began to decline after the first treatment cycle and continued to decline significantly after the second treatment cycle (Fig. 6c and d), demonstrating the alleviation of inflammation after pamidronate disodium treatment. The levels of bone formation marker, serum osteocalcin, were lower after the second treatment cycle (Fig. 6e). Following the first treatment, serum β-CTx levels decreased significantly, and there is a slight but not significant increase after the second treatment (Fig. 6f). Notably, during the treatment period, levels of serum Sp17 autoantibodies in the sera of SAPHO patients decreased continuously (Fig. 6g), which further confirmed that the Sp17 autoantibody levels were correlated strongly with the disease activity and inflammatory status of patients with SAPHO syndrome.

## Discussion

The major finding of this study is the identification of an anti-Sp17 autoantibody as a potential biomarker for the diagnosis and monitoring of disease activity in patients with SAPHO syndrome. Although the pathogenesis of SAPHO syndrome is multifactorial, immunological dysfunction plays a crucial role in its development [19]. Total IgG levels in the sera from patients with SAPHO syndrome were elevated compared to HCs, indicating the excessive activation of humoral immunity in the patients with SAPHO syndrome. This finding is consistent with previous findings that SAPHO syndrome is accompanied by remarkably elevated serum IgG4 levels [20]. We then screened the profile of autoantibodies in the sera of SAPHO patients by using protein chips containing 17,000 human proteins, and two specific autoantibodies against Sp17 and UACA in the sera of SAPHO patients were detected. However, only the anti-Sp17 autoantibody was confirmed in sera from a larger group of SAPHO patients by using ELISA and western blot assays; in contrast, levels of anti-UACA autoantibodies were undetectable by both ELISA and western blot assays. Thus, anti-UACA autoantibodies are not suitable for development as markers.

Sp17 autoantibody levels in the sera from patients with SAPHO syndrome are associated with disease activity, which was confirmed by a correlation analysis of Sp17 autoantibodies with two inflammatory markers: hsCRP and ESR. Indeed, serum levels of Sp17 autoantibodies exhibited a significant positive correlation with serum levels of hsCRP and ESR in patients with active SAPHO syndrome. Importantly, the serum anti-Sp17 autoantibody level may be a better specific marker for the early diagnosis or monitoring of disease activity in patients with SAPHO syndrome than serum hsCRP and ESR levels. In fact, serum hsCRP and ESR levels are elevated in patients with many inflammatory diseases and immunological disorders [21, 22], but elevated serum levels of anti-Sp17 autoantibodies have not been reported in patients with any other autoimmune diseases.

Sp17 autoantibody levels in the sera from patients with SAPHO syndrome are associated with bone metabolism status, which was confirmed by a correlation analysis of Sp17 autoantibodies with two bone metabolism markers: osteocalcin and β-CTx. Importantly, osteocalcin is a bone-specific calcium-binding protein released during bone formation and resorption by osteoblasts. β-CTx is the main fragment of the type I collagen degradation by osteoclasts. An elevated level means that there is osteolysis and strong bone resorption. Indeed, serum levels of Sp17 autoantibodies exhibited a significant positive correlation with serum levels of osteocalcin and β-CTx in patients with active SAPHO syndrome, which suggested serum levels of Sp17 autoantibodies are associated with osteoarthritis and osteolytic lesions.

Sp17 is a highly conserved mammalian protein, and based on early studies, it is widely believed to be a testis-specific protein that is expressed at high levels during the sperm acrosome reaction. Jong et al. [23] further validated the distribution of Sp17 isoforms in various tissues using RT-PCR and detected the *Sp17-1a* mRNA in the human adrenal glands, lymph nodes, skeletal muscle, spine, ovary, and adult testis, whereas esophageal *Sp17-1a* and *Sp17-1b* mRNAs have both been detected in PBMCs, the parathyroid gland, and the synovium. Sp17 was recently reported to be a highly immunogenic protein, and Sp17 autoantibodies have been detected in vasectomized men [24] and patients with periampullary carcinoma [25]. The findings of the present study provide new insights into the potential pathogenesis of SAPHO. Therefore, further studies should be performed to explore the mechanisms underlying Sp17 targeting by the immune system and its roles in the pathogenesis of SAPHO syndrome.

The key future characteristics of patients with SAPHO syndrome are inflammatory skin and osteoarticular manifestations. Multiple immunosuppressive drugs aiming to alleviate inflammatory symptoms have been used. Nonsteroidal anti-inflammatory drugs (NSAIDs) are commonly used to relieve pain and skeletal injuries. However, in most cases, the effects are transient and the disease relapses after NSAID withdrawal [26]. As a therapeutic option for SAPHO cases that are unresponsive or refractory to conventional drugs, biological inhibitors targeting the inflammatory mediators IL-1 and TNF-α are effective at improving bone, skin, and joint manifestations. However, populations benefiting from this treatment regimen have not been clearly identified. Patients with a worsening disease or who are unresponsive to anti-TNF-α drugs have been reported [5]. Because of the inhibition of bone resorption and the anti-inflammatory effect, some studies have used bisphosphonates to control inflammation and pain related to bone resorption. Bisphosphonates significantly and rapidly relieve symptoms in patients with SAPHO syndrome and exert a long-term effect on inflammation and spinal bone marrow edema, which is strongly correlated with musculoskeletal pain [7]. Similar results were obtained in our study, in which VAS, hsCRP, and ESR levels were markedly decreased after pamidronate treatments. β-CTx showed a significant decrease after the first treatments, while the osteocalcin declined until the second treatment cycle compared with the baseline. Notably, during the treatment period, serum levels of Sp17 autoantibodies decreased continuously in patients with SAPHO syndrome. Serum Sp17 autoantibody was more sensitive for the efficacy of bisphosphonate treatments in SAPHO syndrome than β-CTx and osteocalcin, which further confirmed that the level correlated strongly with the disease activity and inflammatory status of patients with SAPHO syndrome. 

In summary, our major finding in this study is the identification of the anti-Sp17 autoantibody as a potential biomarker for patients with SAPHO syndrome. This finding may also provide us with a novel clue for exploring the pathogenesis of SAPHO syndrome.

## Supplementary Information

Supplementary figure 1 **Correlation between levels of ESR and serum IgG.** The Spearman correlation was used to analyze correlations. *n* = 45 (PNG 58 kb)

High Resolution (TIF 2815 kb)

## Data Availability

All data used to support the findings are included in the article and are available from the corresponding author upon request.
