# Supplementary figures and images for: Serum Sp17 Autoantibody Serves as a Potential Specific Biomarker in Patients with SAPHO Syndrome
Source: J Clin Immunol. 2021 Jan 3;41(3):565–75. doi: 10.1007/s10875-020-00937-w (PMC7921076; doi:10.1007/s10875-020-00937-w)

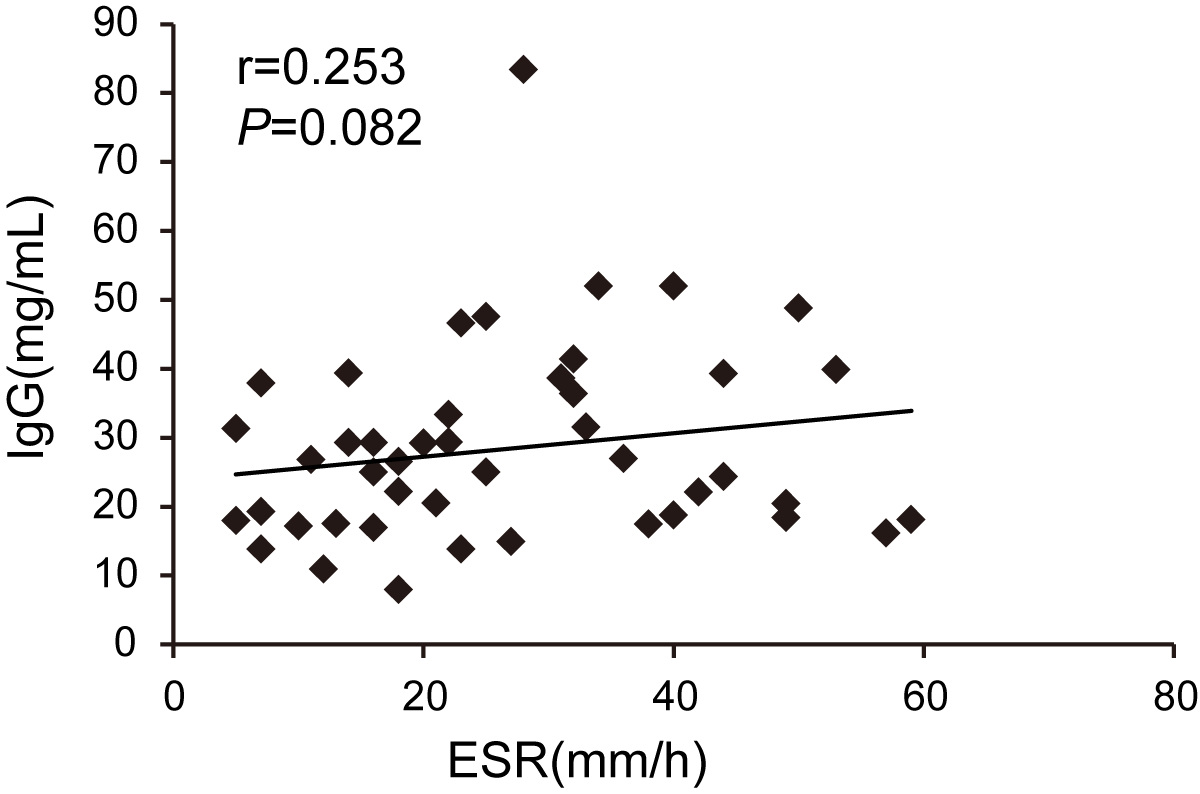

Supplement: Supplementary file 1 — Correlation between levels of ESR and serum IgG. The Spearman correlation was used to analyze correlations. n = 45 (PNG 58 kb) [file 10875_2020_937_Fig7_ESM.png]

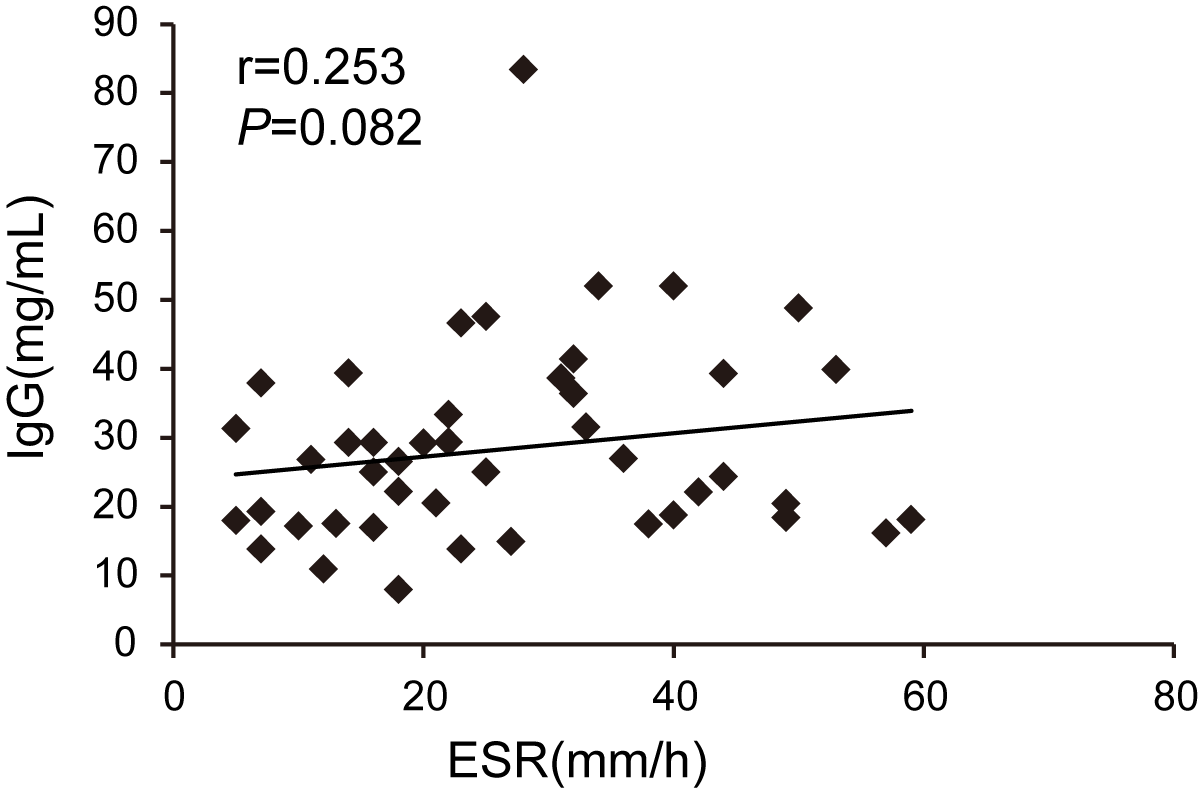

Supplement: Supplementary file 2 — High Resolution (TIF 2815 kb) [file 10875_2020_937_MOESM1_ESM.tif]
